# Minimally Invasive Approach for Improving Anterior Dental Aesthetics: Case Report with 1-Year Follow-Up

**DOI:** 10.1155/2018/4601795

**Published:** 2018-09-06

**Authors:** H. Sevilay Bahadır, Gökhan Karadağ, Yusuf Bayraktar

**Affiliations:** Kırıkkale University Faculty of Dentistry, Department of Restorative Dentistry, Turkey

## Abstract

Dental aesthetics have become highly important in recent years. Treating aesthetic demands with noninvasive or minimally invasive techniques can preserve the natural tissues. A 20-year-old female patient presented to the clinic with aesthetic concerns. After the clinical and radiographic examinations, hypomineralization was identified in the maxillary anterior teeth except the maxillary right canine. An external discoloration was also identified in the maxillary left canine tooth. Moreover, the right canine tooth was identified as a Turner's tooth according to the patient's anamnesis. The resin infiltration technique was applied to the maxillary anterior teeth except the maxillary right canine. The bleaching treatment was applied to the maxillary left canine tooth. Then, a laminate veneer restoration was applied to the upper right canine tooth with Turner's hypoplasia. Following the treatment, a satisfactory aesthetic restoration was achieved. After 1-year examination, no clinical failures were observed.

## 1. Introduction

Aesthetic dentistry has recently gained popularity with the aesthetic factors becoming highly important. More patients seek a visually pleasing smile, and the perception in the media about the concept of beauty has improved. Nowadays, patients' demands for invisible restorations performing with minimal invasive applications to dental tissues which provide a natural look have increased. Advanced restorative techniques along with biomimetic materials and the philosophy of preventive dentistry support the regaining of healthy, functional, and aesthetic smiles [1].

Tooth discolorations, hypocalcifications, and surface irregularities are important aesthetic concerns [2]. Tooth discolorations vary in etiology, appearance, localization, and tooth structure. They are classified as internal, external, and a combination of both. Vital bleaching treatment has been accepted for discoloration treatment [3].

Also, developmental defects of the enamel are important aesthetic concerns. Developmental defects of the enamel are basically classified under two main categories: enamel hypoplasia and enamel hypomineralization caused by an insult to the ameloblasts during amelogenesis. Hypomineralization (opacity) is a qualitative developmental defect of the enamel caused by incomplete enamel mineralization and maturation below the enamel surface that is intact at the time of eruption. The defect reveals a variable degree of alteration in the translucency of the enamel, which has initially a normal thickness and can be white, yellow, or brown. The border of the defect can be demarcated [4–6]. Hypoplasia is characterized as the decreased thickness of the enamel to varying degrees and pit and surface irregularities. Although the transparency and hardness of the enamel remain the same, the opacities may vary from small to large [2].

Turner's hypoplasia is a type of hypoplasia characterized by imperfections in a permanent tooth caused by trauma or periapical infections of deciduous teeth and commonly irritate patients aesthetically. The degree of hypoplasia can vary from light brown color to darker shades in the affected area. It may also affect the whole crown. This is often observed in maxillary incisors and maxillary and mandibular molar teeth [7].

“Dental fluorosis,” a specific disturbance in tooth formation and an aesthetic condition, is defined as a chronic fluoride-induced condition, in which enamel development is disrupted, and the enamel is hypomineralized. Clinically, enamel fluorosis is seen as white spots, brown stains, white opaque lines or striations, or a white parchment-like appearance of the tooth surface. Fluorosis is symmetrically distributed, but the severity varies among the different types of teeth. Teeth such as premolars have a higher prevalence of fluorosis and are more severely affected [8].

Many aesthetic treatments are used for these aesthetic imperfections. Problems such as Turner's hypoplasia, fluorosis, and external factors are usually treated with methods including bleaching, microabrasion technique, and laminate veneer restoration. The selection of the optimal treatment technique is related to the degree of discoloration [9].

This case report is aimed at reporting the treatment of a 20-year-old female patient with aesthetic concerns in the anterior teeth using a minimally invasive approach and evaluating the clinical performance after 1 year.

## 2. Case Report

**Figure 1:**
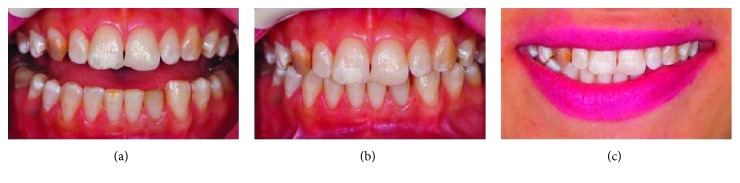
(a) Appearance of teeth in the half-open position. (b) Position of teeth in occlusion. (c) Lip position during a smile, smile line, and teeth visibility.

A 20-year-old female patient was admitted to the Department of Restorative Dentistry, Faculty of Dental Medicine, Kırıkkale University, with aesthetic concerns. The anamnesis did not specify any systemic illnesses of the patient. After the clinical and radiographic examinations, hypomineralization was identified in the maxillary anterior teeth except the right canine tooth. An external discoloration was also identified in the left canine tooth. Moreover, the right canine tooth was identified as a Turner's tooth according to the patient's anamnesis. (Figure 1).

**Figure 2:**
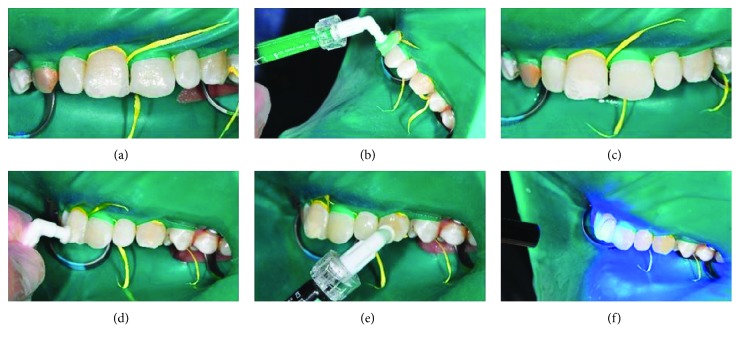
(a) Application of the acid gel. (b) Washing with a water spray. (c) Application of an ethanol drier. (d) Application of a low-viscosity resin infiltrate. (e) Polymerization. (f) Immediately after application.

A minimally invasive and aesthetically satisfactory treatment plan was made with the consent of the patient. The resin infiltration technique (Icon, DMG, Hamburg, Germany) was applied to the maxillary anterior teeth except the right canine. A rubber dam was implemented to protect the soft tissues and create a clean and dry working environment. The teeth were then cleaned with a cleaning pad, and the resin infiltration technique was applied step by step as follows. (1) A gel comprising 15% HCl, water, silica, and other additives was applied for 2 min with a special apparatus to ensure its homogenous application. Then, the acid gel was washed with a water spray for 30 s. (2) An ethanol drier was applied for 30 s to remove water in the lesion area and make the microporosity of the enamel surface more visible. (3) Finally, a low-viscosity resin infiltrate was applied for 3 min. Excess materials were removed with a cotton roll and a dental floss. Finally, a light curing accessory was used for polymerization and polishing for 40 s. (Figure 2).

**Figure 3:**
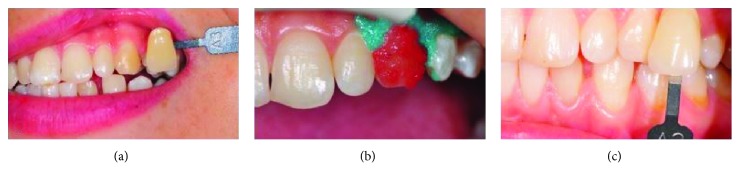
(a) Tooth was determined as A3 on the Vita scale. (b) Bleaching agent was applied. (c) Tooth was determined as A2 on the Vita scale.

Later on, the left canine tooth was identified to have a darker color and hence bleaching treatment was applied. First, the color of the left canine tooth was determined as A3 on the Vita scale. Then, a gingival protective gel was applied to the contours of the gums following the manufacturer's protocol and polymerized with an LED light accessory. Two components of the whitening agent (Opalescence Boost PF, Ultradent, YT, USA) were mixed following the manufacturer's protocol and applied to the aforementioned area. The application took 40 min in one session, and the color of the tooth was determined as A2 on the Vita scale (Figure 3).

**Figure 4:**
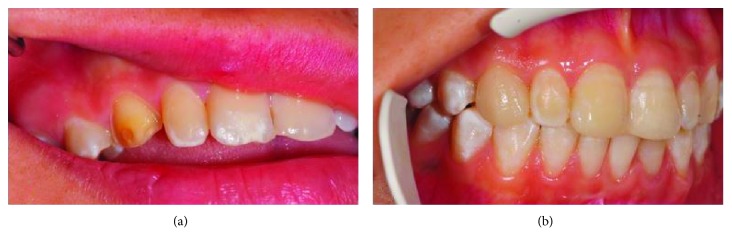
(a) Overlapped preparation. (b) Application of laminate veneer.

A laminate veneer restoration was planned for the upper right canine, which was a Turner's tooth according to the patient's anamnesis. The tooth was prepared (Komet Ceramic Veneer Kit, Komet Dental, Hamburg, Germany). The overlapped preparation was applied to exceed the incisal enamel contours by 2 mm. The gingival retraction was achieved using a combination of mechanical and chemical retraction methods. The prepared tooth and the opposite teeth were digitally measured with an intraoral scanner (TRIOS A/S, 3Shape Trios, Copenhagen, Denmark). The designed laminate veneer was produced with a CAM system (Coritec 550i, imes-icore, Eiterfeld, Germany) using lithium disilicate glass ceramic blocks (IPS e-max CAD, Ivoclar Vivadent, Schaan, Liechtenstein). The restorations were glazed in the laboratory and then cemented with adhesive cement (Panavia V5 Clear, Kuraray Noritake Dental, Tokyo, Japan) following the recommendations of the manufacturer (Figure 4).

**Figure 5:**
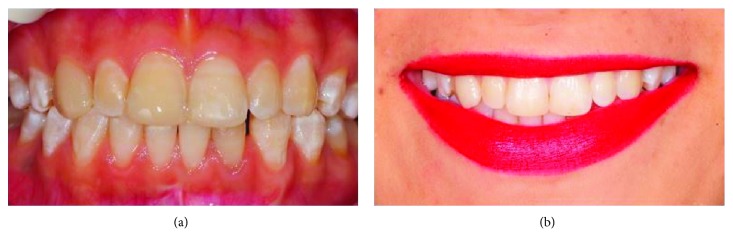
(a) Posttreatment. (b) One-year follow-up.

Incisal irregularities on the maxillary central incisors were restored with a nanohybrid composite resin (Filtek Ultimate A2 Body, 3M ESPE, St. Paul, MN, USA). Following the treatment, a satisfactory aesthetic restoration was achieved. The examination of the restorations after 1 year did not reveal any clinical failures (Figure 5).

## 3. Discussion

Developmental defects of the enamel are caused by various etiological factors, such as amelogenesis imperfecta, overexposure to fluoride along the mineralization of the enamel, different disorders, or trauma. However, their origin is often unknown. Hypomineralization can occur independently or coexist with hypoplasia in one or more teeth depending on time, duration, susceptibility of the individual, and severity of the prenatal, perinatal, or postnatal insult [4, 6]. In this case report, the patient did not report any possible cause for the hypomineralization.

Reestablishing the visual dental aesthetics of a patient has been one of the chief purposes of modern dental medicine. Novel materials and treatment methods are being developed every day to reach this goal [10].

The resin infiltration technique is one of the methods for addressing aesthetic demands. Kielbassa et al. and Paris et al. first developed and applied this technique to cure proximal caries, which demonstrated the same grading level in histological caries extension [11, 12]. Later, this technique was used for white spot lesions and demonstrated to remove the white stains on the enamel [11, 13]. It was further implemented in cases of hypoplasia and fluorosis [2]. Defects such as white spot lesions, hypomineralization, and hypoplasia exhibit opacity because refractive indices of enamel crystals and the insides of the pores are different. The micropores of these lesions are filled with water or air. The resin infiltration technique infuses these micropores with a low-viscosity resin. Thus, the difference in the refractive indices between the micropores and the enamel is eliminated, and the lesion gains an enamel-like appearance [2, 14].

In this case report, the resin infiltration technique was applied to the maxillary anterior teeth except the right canine, all of which exhibited hypomineralization. The procedure yielded aesthetically satisfactory results, corroborating the findings of previous studies [2, 14–16]. Only the maxillary anterior six teeth were restored, as desired by the patient. The reason for the upper right central tooth not responding to the treatment as effectively might be that the thicker and more mineralized surface layers in lesions (pseudointact surface layer or sound enamel) and the pores of the lesion were contaminated with organic materials, such as proteins and carbohydrates, that hampered resin penetration [11, 12].

The resin infiltration technique has some limitations. These limitations are pseudointact surface layer of lesion, mineral content of lesion, amount of micropores of lesion, and structure of roughened surface of lesion. Furthermore, it has not been completely explained whether organic materials, such as biofilm remnants, carbohydrates, lipids, and proteins, attach to the inner enamel surfaces, thus possibly occluding the (underlying) pores in lesions and leading to an incomplete resin penetration of the porous structures. Additionally, deproteinization procedures using sodium hypochlorite should be implemented as procedural prerequisite with the infiltration technique because a cleaned surface would need to assure retention and bond strength to hamper biofilm formation and to impede further cariogenic challenges to the infiltrated lesion and to increase ingress of the infiltrate's resinous matrix [17].

Additionally, bleaching treatment and laminate veneers are methods of addressing aesthetic demands. Bleaching treatment is a more conservative method compared with other methods used for treating discoloration. Office-type whitening treatment is one of the most popular methods. It involves the application of 25%–40% hydrogen peroxide or 16%–35% carbamide peroxide on the external surface of the teeth. The bleaching mechanism works on the principle that hydrogen peroxide penetrates the tooth and generates free radicals that oxidize the organic stains [18]. Several studies [19, 20] in the literature reported that the office-type whitening was successful.

Porcelain laminate veneers are nowadays commonly used for aesthetic purposes owing to their better aesthetic properties, higher resistance to abrasion and discoloration, and better biological harmony with the oral flora [21].

In this study, the porcelain laminate application was used for the upper right canine tooth with a CAD/CAM system. The CAD/CAM technology has improved significantly in clinical applications. It has been commonly used in the practical dental applications. Using this method, satisfactory aesthetic and functional results have been obtained in a short time, making the lives of both the patient and the dentist easier.

Composite resins or porcelain materials are generally preferred materials for aesthetic procedures. In this study, lithium disilicate glass ceramic material was used. Besides its satisfactory aesthetic quality, it also has high endurance against stretching, breaking, and chemicals. The rate of abrasion of the opposite teeth is lower, and the material has higher transparency compared with all other porcelain types [21, 22].

Nowadays, restorative treatment has achieved high aesthetic standards. It protects the dental structure maximally thanks to the development of adhesive systems, resin cement, and ceramics. The resin infiltration technique, bleaching treatment, and laminate veneer applications, among other minimally invasive treatments, have gained importance due to greater protection rates of the tooth and high aesthetic standards.

## 4. Conclusions

The resin infiltration technique, bleaching treatment, and laminate veneer treatment have been shown to be highly conservative methods that bring back a healthy and harmonious smile. The patient discussed in this case report was aesthetically and functionally satisfied with the treatment after 1-year follow-up.

## Additional Points

*Clinical Significance*. Bleaching and resin infiltration techniques are noninvasive techniques for aesthetic dentistry. If these techniques are used in relevant cases, aesthetic results can be achieved.
